# Differences between auto-fluorescence and tetracycline-fluorescence in medication-related osteonecrosis of the jaw—a preclinical proof of concept study in the mini-pig

**DOI:** 10.1007/s00784-020-03332-2

**Published:** 2020-05-22

**Authors:** Oliver Ristow, Dirk Nehrbass, Stephan Zeiter, Daniel Arens, Julius Moratin, Christoph Pautke, Jürgen Hoffmann, Christian Freudlsperger, Sven Otto

**Affiliations:** 1 Department of Oral and Maxillofacial Surgery, University Hospital Heidelberg, Im Neuenheimer Feld 400, D-69120 Heidelberg, Germany; 2 AO Research Institute Davos, Clavadelerstr. 8, 7270 Davos, Switzerland; 3 Department of Oral and Maxillofacial Surgery, Ludwig-Maximilians University of Munich, Lindwurmstr. 2a, D-80336 Munich, Germany

**Keywords:** MRONJ, BRONJ, AR-ONJ, Therapy, Animal model, Fluorescence-guided bone surgery

## Abstract

**Objectives:**

Fluorescence-guided bone surgery is a well-established technique in the treatment of medication-related osteonecrosis of the jaw. No histopathological evidence for bone auto-fluorescence is currently available, and thus, any differences from tetracycline-fluorescence remain unclear. Therefore, the goals of this study were to find out if macroscopic and histological differences occur between the auto- and tetracycline-fluorescence in the delineation of viable and necrotic jawbone in the mini-pig.

**Materials and methods:**

According to the proof of concept, osteonecrosis was provoked in eight Göttingen minipigs. Pigs were divided into two groups (AF group: no fluorochrome label; TF group: tetracycline label). Delineation of necrosis and viable bone was evaluated in vivo and in vitro macro−/microscopically, correlated to fluorescence properties and compared between the two study groups.

**Results:**

No macroscopic and microscopic clinical differences were seen in fluorescence between the AF and TF groups. Macroscopic and microscopic viable bone fluoresced green, whereas necrotic bone showed no or only pale fluorescence in both groups. The auto-fluorescence was attributable to the arrangements and structure of collagen and the cell-filled bone lacunae.

**Conclusion:**

Neither in vivo nor in vitro macroscopically differences are apparent between the auto-fluorescence and the tetracycline-fluorescence of bone. The auto-fluorescence is attributable to the arrangements and structure of collagen and the cell-filled bone lacunae. Tetracycline-fluorescence is a mixture of tetracycline (at the bone edges with increased bone formation) and large components of auto-fluorescence.

**Clinical relevance:**

Because auto-fluorescence is easy to apply, reproducible, and does not rely on the subjective impression of the surgeon, it promises to be an important standardized alternative to tetracycline-labeled MRONJ therapy.

## Introduction

Because of their key role in the management of osteoporosis and metastatic bone diseases, the intake of antiresorptive drugs is rapidly increasing worldwide [1–4]. Unfortunately, their major side effect, namely the medication-related osteonecrosis of the jaw (MRONJ), has become a serious problem in oral and maxillofacial specialties. Thus, jaw osteonecrosis has become a key clinical and research focus, particularly with respect to establishing therapeutic standards for the disease.

Regardless of the surgical techniques applied to eliminate necrotic bone, the challenge and limitations of treatment are always the exact determination of the osteonecrosis margins. Surgical experience supported by various imaging modalities is used to resect as much necrotic tissue as necessary and as little as possible healthy bone [5]. Nevertheless, surgical therapy is dependent on the subjective surgeon’s impression of the case under consideration, which is an aspect that is neither comparable nor reproducible.

It has been demonstrated that tetracycline-fluorescence-induced bone fluorescence and fluorescence-guided bone surgery are important tools in the surgical management of MRONJ, as they successfully address the above shortcoming [6–11]. The fluorescence technique provides an objectified and reproducible therapeutic approach and enables the transitions between necrotic and non-necrotic bone to be defined during surgical procedures.

Reports suggest that the VELscope Vx system® (LED Dental, White Rock, British Columbia, Canada) induces an auto-fluorescence of vital (but not of necrotic) bone leading to bone fluorescence findings that are similar to those of tetracycline labeling, which can therefore be omitted. In preliminary investigations, promising results have been obtained concerning success rates following this technique, verifying the complete removal of the necrotic bone as seen in histological preparations [12]. Thus, good outcomes were reported in the first clinical trials [13]. As a consequence, the auto-fluorescence of bone in surgical MRONJ therapy has become an alternative to the use of tetracycline-fluorescence-guided bone surgery.

However, because of the lack of basic research, no definitive evidence exists establishing that bone auto-fluorescence is a histopathological sign of viable bone and non-fluorescence an indication of necrotic bone. Furthermore, any differences from tetracycline-fluorescence remain unclear. Justifiably, one might argue that what was assumed to be tetracycline and auto-fluorescence over the last decade was rather a mixture of the tetracycline- and auto-fluorescence of bone.

In order to eliminate this shortcoming, the objective of this study was to use the previously implemented and optimized large animal model of the authors, namely the minipig [14, 15]. The major goals of this preclinical study were (i) to establish the auto-fluorescence of healthy bone (no auto-fluorescence in necrotic bone), (ii) to investigate the histopathological setup and the reason for the auto-fluorescence, and (iii) to compare the macroscopic and histological differences between auto-fluorescence and tetracycline-fluorescence. The main objective was to find out if there are macroscopic and histological differences between the auto- and tetracycline-fluorescence in the delineation of viable and necrotic jawbone in the mini-pig.

## Materials and methods

### Animals

The study was carried out at the AO Research Institute (ARI) in Davos, Switzerland, and performed according to Swiss laws of animal welfare. The study was approved by the cantonal Animal Welfare Commission (authorization number: 28/2016) of Grisons (Switzerland) and conducted in an AAALAC International approved facility. After approval from the authorities, eight female Göttingen minipigs (Ellegaard, Denmark) were delivered to ARI (animals were 15 months of age and had an average bodyweight at the start of the study of 42.5 kg (range of 39 to 45 kg). All animals were healthy based on clinical examination by a veterinarian. Before the start of the experiment, the animals underwent an acclimatization period of 7 weeks. During this time, feeding was performed partly by hand to aid in post-operative handling. The pigs were trained with regard to the procedures carried out during the experiment (e.g. weight measurement, blood withdrawal) and were housed in groups on deep straw with access to the outside. Animals were fed with pellets (Maintenance feed, Art 3000, Provimi Kliba AG, Switzerland).

### Phase one: large animal model

After acclimatization, all animals received antiresorptive treatment of zoledronate (0.05 mg/kg) intravenously once a week for the 12 subsequent weeks. The weekly zoledronate injections and all examinations of the oral cavity were performed under deep sedation. The treatment went ahead as planned, without any incidences or complications.

**Fig. 1:**
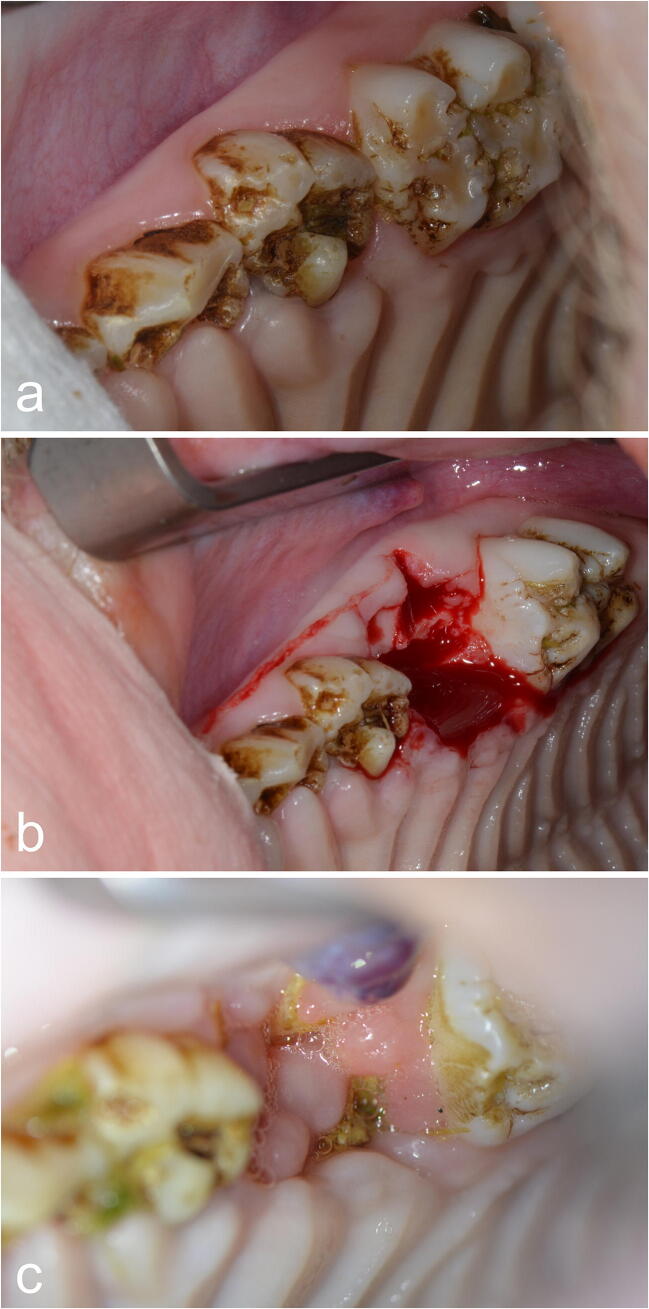
TF group, intervention side: first surgical intervention after 12 weeks of antiresorptive pretreatment. Left maxilla of a Göttingen minipig **a** before and **b** after extraction of the first molar. **c** Exposed bone in the extracted area 8 weeks later

The first surgical intervention was performed after the above pretreatment of 12 weeks at ARI. All surgical procedures were performed under general anesthesia according to our standardized protocol [15]. The first molar teeth of the mandible and the maxilla were extracted unilaterally (Fig. 1) to determine whether any differences occurred between the upper and lower jaw. On the contralateral side, no teeth were extracted, which was therefore used as the control side. All tooth extractions were performed without incidences or complications, apart from some small root remnants.

After surgery, antiresorptive treatment was continued for the subsequent 8 weeks (0.05 mg/kg intravenously once weekly). The weekly Zoledronate injections and examinations of the oral cavity were again performed under deep sedation, without any incidences or complications. Regular examinations for clinical signs of oral lesions were performed throughout the study and photo-documented (Fig. 1). Ten days before death, the animals were randomly assigned to two groups. Fluorescent-labeling of the bone with tetracycline (25 mg/kg intravenously once a day) was performed in half of the animals (TF group, *n* = 4), whereas the other half received no fluorochrome labeling (AF group, *n* = 4).

**Fig. 2:**
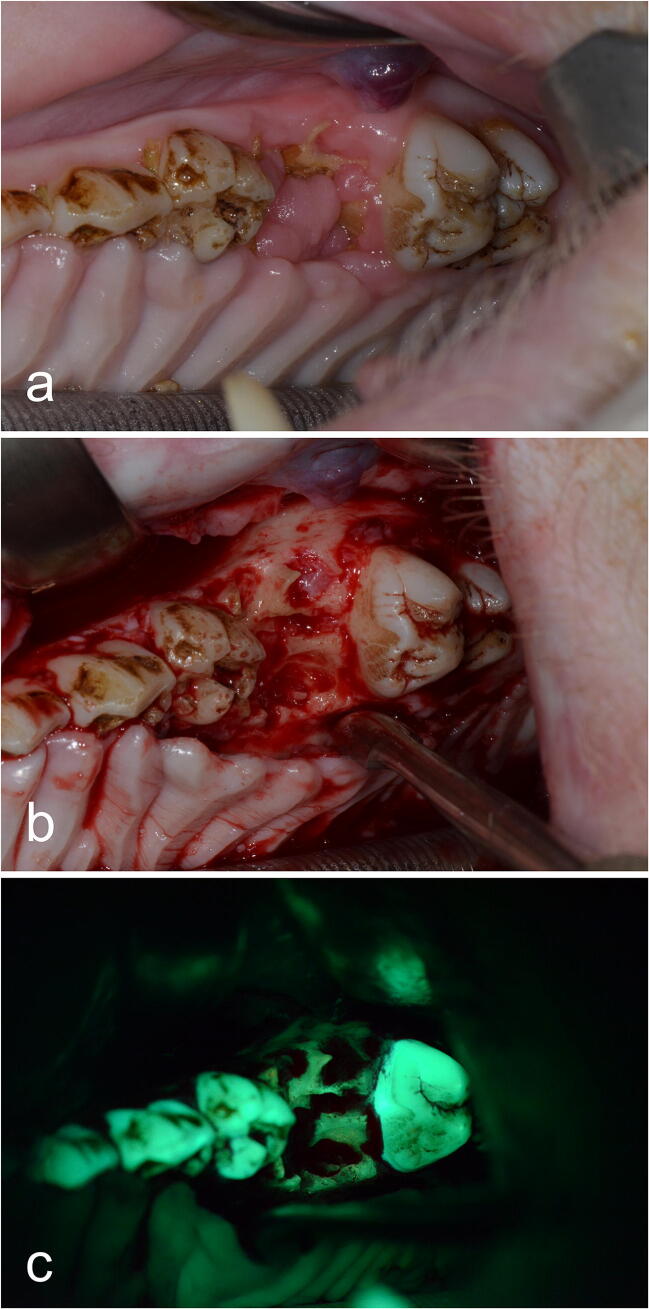
TF group, intervention side: **a** second surgical intervention 8 weeks after first surgical intervention. Necrosis and viable areas following in vivo sub-periosteal preparation **b** without and **c** with fluorescence light

The second surgical intervention was performed 8 weeks after the first surgical intervention and after a continuation of the antiresorptive treatment for another 8 weeks. All surgical procedures were performed under general anesthesia according to our standardized protocol [15]. Following in vivo sub-periosteal preparation of the MRONJ regions (and of the control side), detailed high-resolution photographic documentation was performed on macroscopic necrosis signs and on non-diseased areas (with and without illumination by fluorescence with the VELscope Vx system® (blue excitation light: spectrum 400–460 nm and a green filter with an emission open from 460 nm)) (Fig. 2). This enabled subsequent planned data analysis, correlating in vivo and in vitro macroscopic and histological results.

### Phase two: post-mortem and macroscopic preparations

**Fig. 3:**
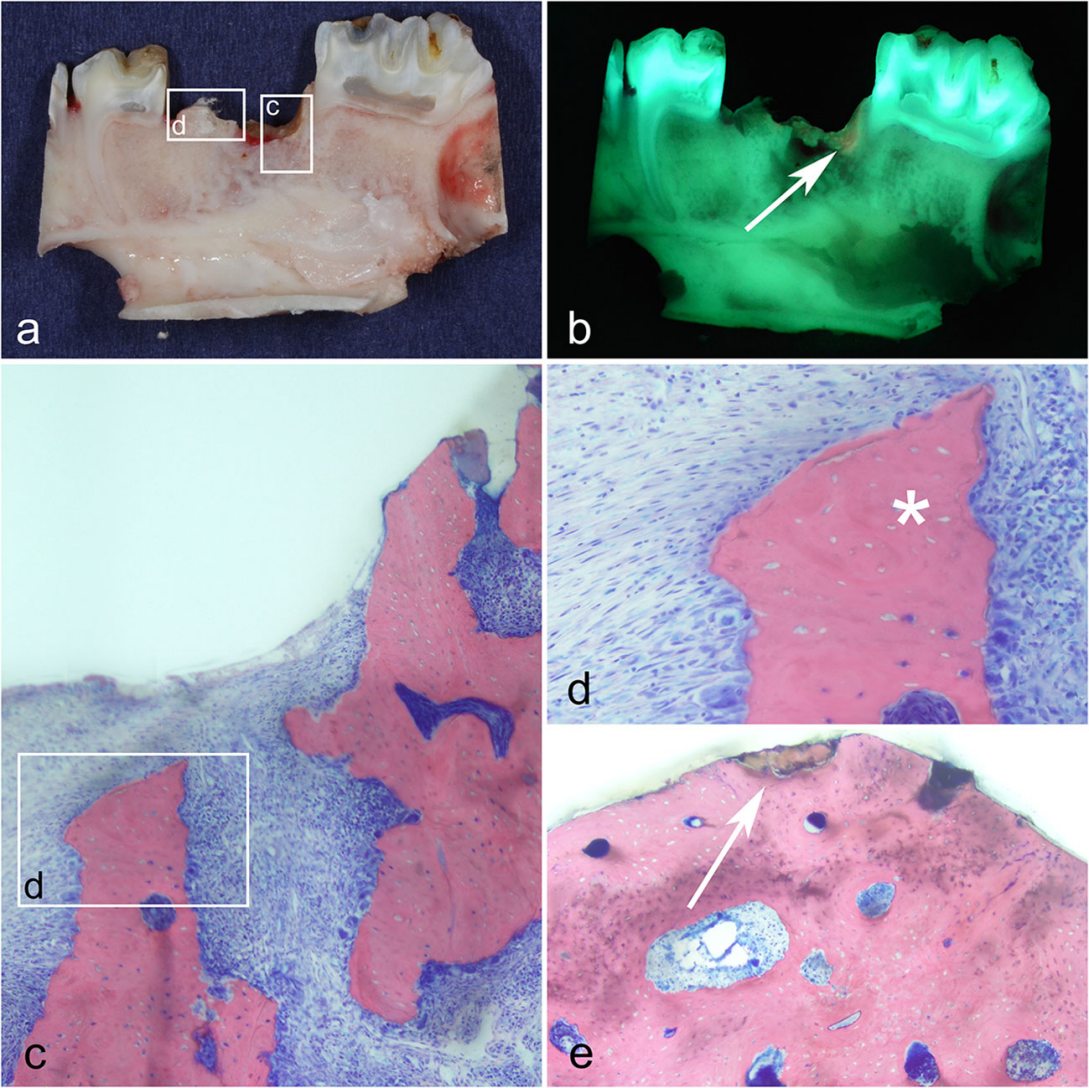
TF group, intervention side: post-mortem-prepared macroscopic bone blocks in a sagittal plane. **a** Macroscopic delineation between necrotic and viable bone. **b** Corresponding fluorescence view. Arrows indicates necrotic bone. **c** Corresponding histopathologic Giemsa-Eosin-stained sections **d** early necrotic bone lesion with empty osteocytic lacunae (*) at bone stock. **e** Demineralization of extracellular bone matrix (associated with denuded bone) (arrow)

Directly after death, the mandible and maxilla of each pig were harvested for macroscopic and histological in vitro analysis and sent to the laboratory for further preparation. In the laboratory, regions of interest in the upper and lower jaw (intervention and control side) were cut into blocks by using a diamond-coated band saw (CP 310, Exact, Germany). Bone blocks were cut in a sagittal plane for macroscopic in vitro fluorescence acquisition and for preparation for histology. Once the bone blocks had been prepared, they were macroscopically and fluorescently evaluated. In order to enable subsequent data evaluation and mapping in the macroscopic and histopathological preparations, we undertook high resolution photographic documentations (Fig. 3).

### Phase three: histological preparations and examinations

#### Preparation of specimen

Harvested and subsequently midline-sagittally split bone blocks (for every specimen: the upper and lower jaw of the intervention and the control site) were then divided and either prepared as (i) non-decalcified PMMA-embedded (polymerized methyl-methacrylate) sections or (ii) decalcified paraffin-embedded sections.

Sectioning and staining of non-decalcified PMMA-embedded were performed as previously described by our group [15, 16]: bone-blocks were fixed in 70% (*v*/v) methanol for several months, with at least 3 changes of fresh methanol. After fixation, samples of the extraction sites (upper/lower jaw) and from the control site (upper/lower jaw) were trimmed down with a butcher’s saw (Bizerba FK 22, Bizerba AG, Switzerland), dehydrated through an ascending series of ethanol, transferred to xylene, and finally infiltrated and embedded in PMMA. PMMA blocks were cut with a diamond blade saw (CP 310, Exact, Germany) again in the sagittal plane. Sections were glued onto opaque plexiglass holders, ground, and finely polished down to 100 (± 20) μm thickness. Subsequently, half of the sections were stained with Giemsa-Eosin, whereas the other half (for fluorescence analysis) were kept unstained.

In a second step, the opposite site of every bone block was used for the preparation of decalcified paraffin-embedded for each region of interest [14]. The harvested blocks were fixed in buffered 4% formaldehyde at 4 °C for 48 h and then parted and decalcified with EDTA at 25 °C for 28 days, followed by a rinse with water. After dehydration in ethanol, all samples were degreased in xylene and embedded in paraffin. Sections (4 μm thick) were cut by means of a microtome (CP 310, Exact, Germany). Again, half of the sections were histologically stained with Giemsa-Eosin, whereas the other half remained unstained for fluorescence analysis.

#### Histological evaluation and characterization

As a primary outcome, a descriptive analysis was performed after staining in order to depict the necrotic and healthy bone parts in the stained PMMA sections; these results were correlated with the macroscopic fluorescence findings (in vivo data: phase one, and in vitro data: phase two) of necrotic and healthy bone (score: 6 regions of interest per section). Of note, necrosis was expected in the macroscopically non-fluorescent areas in the auto-fluorescence and the tetracycline-fluorescence groups, whereas healthy bone was expected in the fluorescence areas. Evaluation was based on the established and introduced histological characterization [15, 16].

### Phase four: fluorescence microscopy

After confirming the results of the macroscopic fluorescence findings with the histological characterization, fluorescence evaluation was performed directly on the histological sections. A conventional fluorescence microscope (Olympus, Shinjuku, Tokio, Japan) was used with the necessary specifications for the fluorescence of bone and the VELscope Vx system® (individualized filter system U-MNBV: blue excitation light, spectrum 420–455 nm and a green filter with an emission open from 470 nm). Semi-quantitative and descriptive analyses were performed to depict necrosis, and healthy bone signs histologically and were correlated with the macroscopic clinical bone sections and with the macroscopic bone fluorescence.

#### Semi-quantitative evaluation

Fluorescence analysis was performed on the non-stained PMMA and decalcified paraffin sections and compared with the corresponding stained sections for the orientation for both study groups (intervention and control). Again, evaluation was performed on six random regions of interest. In correlation with the histopathologic results, necrotic and non-necrotic bone was evaluated by using the fluorescence microscope technique by applying a scoring system: grade 1 = no fluorescence, grade 2 = weak fluorescence, grade 3 = medium fluorescence, grade 4 = strong fluorescence, and grade 5 = very strong fluorescence.

#### Descriptive analysis

For the second part of the study, both non-decalcified and decalcified histological preparations were evaluated for qualitative differences in order descriptively to determine whether a histologically detectable difference was apparent between auto-fluorescence and tetracycline-fluorescence, i.e., to establish whether auto-fluorescence was mineral-dependent as shown by changes between the PMMA and decalcified paraffin sections.

#### Statistical analysis

Because of the preliminary proof-of-concept character this study, the sample size estimation was disclaimed. Due to the uncertainty of the parameter estimates merging from this pilot and in order to estimate an effect size for this explorative study, we decided to include 8 Göttingen minipigs (4 animals each group; *n* = 32 sites: intervention sites *n* = 16, control sites *n* = 16). The primary outcome of the current study was the correlation between macroscopic findings and histological results. Mainly, qualitative and semi-quantitative analyses were performed. Semi-quantitative analysis was always performed in 6 regions of interest per section, overall *n* = 192 regions of interest. In order to recommend the dissection of necrotic bone based on auto-fluorescence of the bone, this correlation had to be 100% in all investigated cases. Heterogeneity of data was tested using the Wilcox signed-rank test and showed no difference between both groups. The study groups were compared with each other by using the two-sided independent samples *t* test for differences in means. Due to the large number of parameters that were compared all *p* values are of descriptive manner, and no statistical significance is claimed. All statistical analysis was performed using the IBM SPSS Statistics software (Version 20.0).

## Results

### Large animal model

As expected and demonstrated in previous studies, all animals presented exposed bone and fulfilled the criteria (exposed necrotic bone for 8 weeks, history of antiresorptive intake) for the diagnosis of a MRONJ at the close of phase one.

### Macroscopic in vivo evaluation

No macroscopic clinical differences in fluorescence were seen between the auto-fluorescence and the tetracycline-fluorescence groups. Macroscopically viable bone was marked by green fluorescence and visualized intraoperatively with a VELscope Vx system®. In contrast, necrotic bone showed no or only pale fluorescence in both groups. Because mostly early stages of MRONJ were evaluated, a new aspect was serendipitously added to the evaluation, since early stages have not previously been described by using fluorescence, either preclinically or clinically.

### Macroscopic in vitro evaluation

**Fig. 4:**
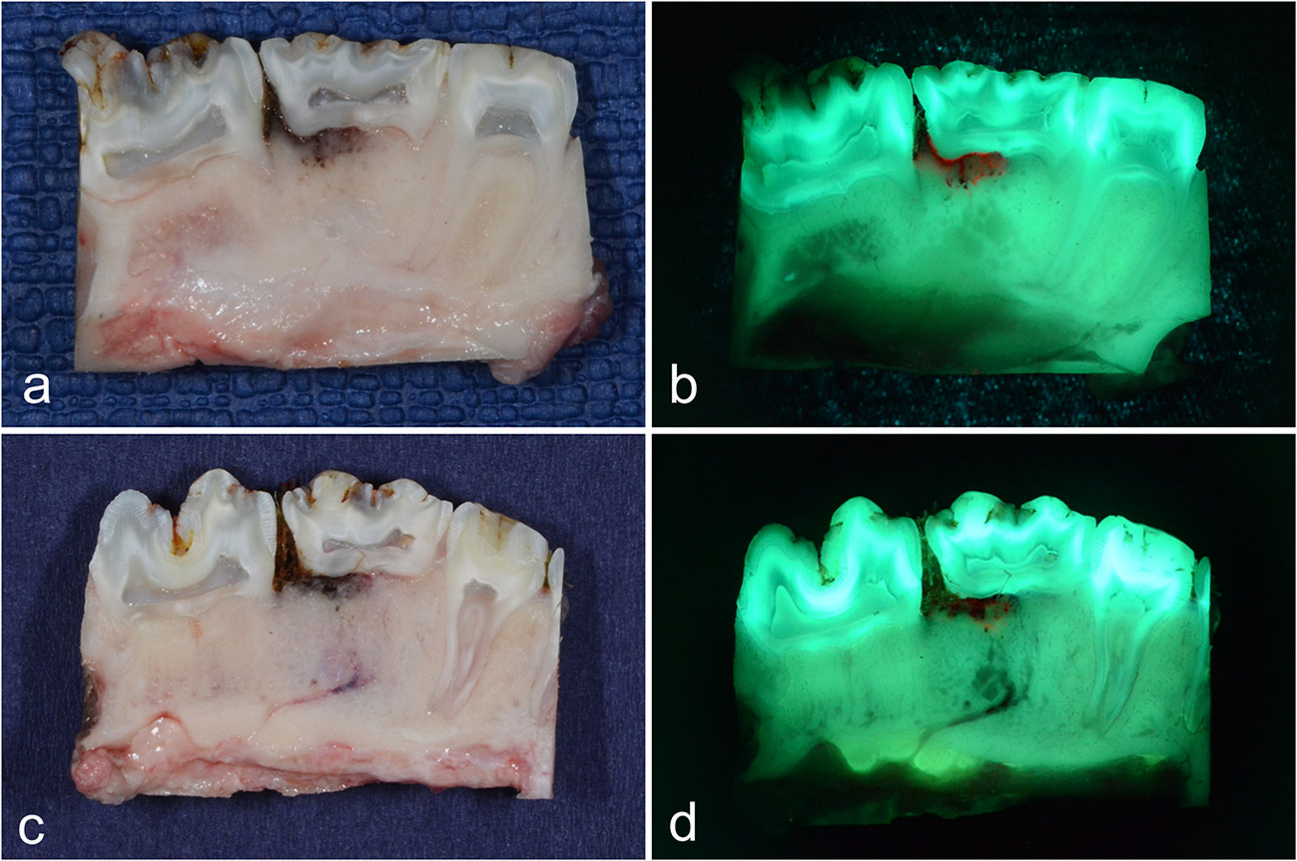
Post-mortem-dissected bone blocks of the AF group (**a** + **b**) and the TF group (**c** + **d**), control side. Necrotic and viable bone areas macroscopically show no differences between the two groups. Thus, viable bone is marked by green fluorescence, and necrotic bone shows no or only pale fluorescence. Note the red fluorescence regions arising from bacterial colonization

Again, neither necrotic nor viable bone areas showed macroscopic differences between the auto-fluorescence and tetracycline-fluorescence groups (Fig. 4). Thus, viable bone was marked by green fluorescence, and necrotic bone showed no or only pale fluorescence in both groups. Interestingly, multiple necrotic lesions were also found in the control areas of the contra-lateral upper/lower jaw control side where food entrapment (therefore, an infectious reaction) was found.

### Histological evaluation and characterization

Macroscopically, fluorescent and non-fluorescent areas were compared with the corresponding histological parts in the stained PMMA sections. In all animals (*n* = 32 sites: intervention sites *n* = 16, control sites *n* = 16), macroscopic fluorescence signs were confirmed (32/32, 100%) in the histological preparations: non-fluorescent areas were confirmed as necrotic bone, whereas green fluorescent areas were histologically confirmed as healthy bone (*n* = 6 regions of interest: per section *n* = 184/192; 96%), affirming the primary outcome of the study. Again, no difference was seen between the auto-fluorescence and the tetracycline-fluorescence groups.

### Fluorescence microscopy

#### Semi-quantitative analysis

Necrosis was found in the non-fluorescent areas in the auto-fluorescence and tetracycline-fluorescence groups (no difference between the two groups). Moreover, healthy bone was found in the fluorescence areas in the auto-fluorescence and the tetracycline fluorescence groups (no difference between the two groups).

The mean fluorescence score for all evaluated regions of interest in all PMMA sections for viable bone was 4.56 (SD ± 0.79) indicating a strong to very strong fluorescence (AF group mean: 4.56, SD ± 0.5; TF group: mean: 4.56, SD ± 1.0; *p* > 0.05). The mean scoring of all evaluated regions of interest in all decalcified paraffin sections was clearly lower at 2.09 (SD ± 0.46) (AF group mean: 2.06, SD ± 0.43; TF group: mean: 2.13, SD ± 0.048; *p* > 0.05). No significant difference was observed with regard to the mean fluorescence between the auto- and the tetracycline-fluorescence groups (*p* > 0.05).

The mean fluorescence score for all evaluated regions of interest in all PMMA sections for necrotic bone was 1.22 (SD ± 0.42) indicating no fluorescence to very weak fluorescence (AF group mean: 1.11, SD ± 0.31; TF group: mean: 1.33, SD ± 0.47; *p* > 0.05). The mean scoring of all evaluated regions of interest in all decalcified paraffin sections was even lower at 1.0 (SD ± 0) (no fluorescence) (AF group mean: 1.0. SD ± 0; TF group: mean: 1.0, SD ± 0; *p* > 0.05). Again, no significant difference was noted with regard to the mean fluorescence between the auto- and the tetracycline-fluorescence groups (*p* > 0.05).

#### Descriptive analysis

**Fig. 5:**
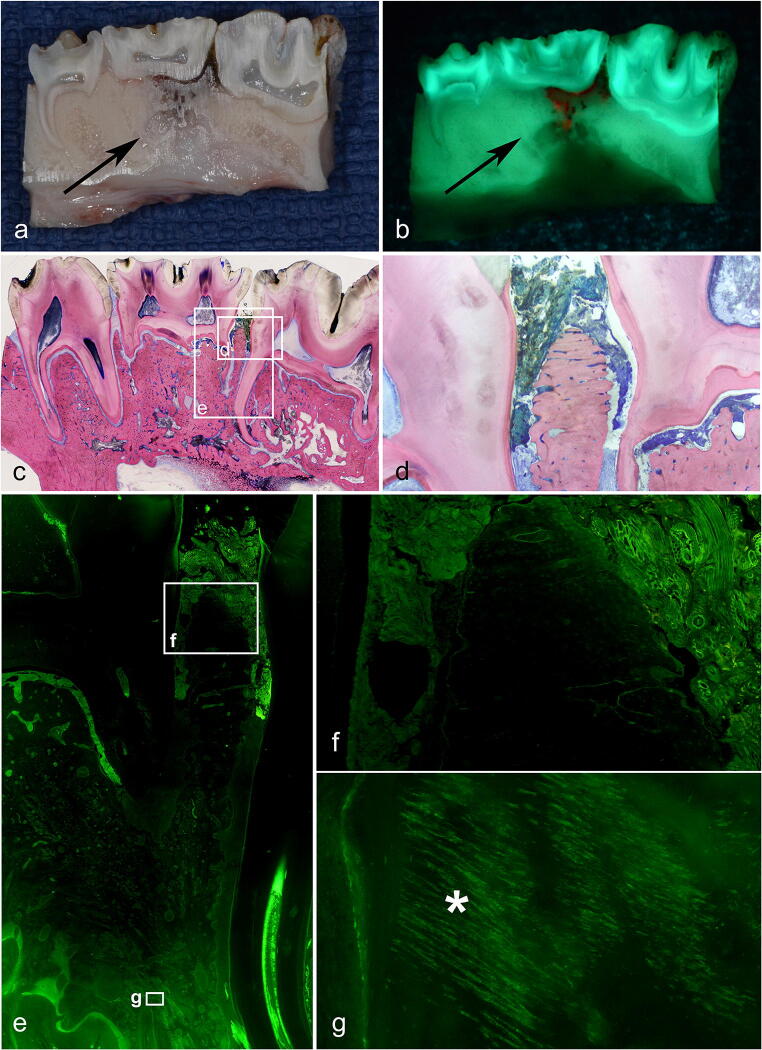
AF group, control side: **a** macroscopic delineation of necrotic bone (arrow). **b** Corresponding necrosis with auto-fluorescence (arrow). Again, note the reddish fluorescence of the bacterial colonization. **c** Overview of the corresponding histopathologic preparation; Giemsa-Eosin-stained sections. **d** Corresponding section in detail. **e** Interdental overview; note the transition from necrotic (no auto-fluorescence, **f**) to viable bone (auto-fluorescence). **g** Note that fluorescence is mainly caused by ordered collagen bands and cells (*)

Histopathologically, a correlation was revealed between the structure of collagen and the fluorescence of the bone. In necrotic areas in which the arrangement and structure of collagen had changed, fluorescence decreased and disappeared. In viable bone in which the collagen remained unchanged, bright fluorescence was observed. In addition to that of the collagen, fluorescence was noted in cell-filled bone lacunae. Therefore, in areas of necrosis (empty bone lacunae), fluorescence was faint (Fig. 5). The correlation between collagen and fluorescence was determined by using a POL filter, which enabled collagen structures to be clearly depicted.

## Discussion

Since necrotic, dead, and infected tissue cannot be resuscitated and interferes with wound healing, surgical therapy is increasingly accepted to be required when necrotic tissue is encountered in the jaw bone [17]. Common sense dictates that as much bone as necessary needs to be eliminated to remove diseased tissue, but as little as possible to spare healthy tissue. However, the determination of the precise margins of the osteonecrosis is difficult [13]. Whereas the complete removal of necrotic bone is of crucial importance, an unintentional or excessive removal of healthy bone must be avoided to ensure that the jawbone is not weakened and to maximize the chances for dental or prosthetic rehabilitation, thereby preserving the quality of life [2, 18].

Although numerous imaging devices help sensitively to detect bone necrosis, no radiologic modality has been available to date having a high specificity for the reliable definition of the extent of the necrosis preoperatively [19]. Arguably, the most reliable parameter is the intraoperative observations of the surgeon, based on the bone appearing to be “normal” in terms of bone structure, color, and texture [20]. Because of its increased porosity, necrotic bone is often softer and surrounded by sclerotic areas, which in turn are harder and less vascularized. Nevertheless, bone bleeding is considered to indicate viable bone, although this has, however, in numerous trials turned out to be a non-reliable indicator for healthy bone [7, 11, 13].

Tetracycline-fluorescence-guided bone surgery represents a solution for this shortcoming [10, 21–23]. The use of tetracycline as a fluorochrome has made it possible, under the appropriate excitation light and filter (VELscope Vx system® fluorescence lamp), to improve the definition of the transition between necrotic (no fluorescence) and non-necrotic (green fluorescence) bone during surgical procedures. Tetracycline, because of its affinity to calcium, was hypothesized to become incorporated into bone predominantly in areas of bone remodeling and bone apposition [24]. Thus, viable bone would fluoresce green, whereas tetracycline would not be incorporated into necrotic bone, which would exhibit no or only pale fluorescence.

Over the years, increasing numbers of reports suggested that the auto-fluorescence of vital (but not of necrotic) bone leads to similar bone fluorescence, even without preceding tetracycline labeling, when using the same fluorescence camera system (VELscope Vx system®) [9, 25]. In a preliminary investigation, our group showed promising results concerning success rates following this technique, verifying the complete removal of the necrotic bone as shown by the histological preparations [12]. In a subsequent randomized clinical trial, we were able to show no inferiority of auto-fluorescence compared with tetracycline-fluorescence for all controlled parameters [13]. Notably, auto-fluorescence seemed to show a graduation in fluorescence: hypo-fluorescence and pale fluorescence to non-fluorescence [13]. Furthermore, this new technique promised additional potential benefits, i.e., non-dependency on patient intake compliance or drug bioavailability and no need for tetracycline administration, with the requirement merely for a single pathologic-spectrum-adapted penicillin antibiotic.

Unfortunately, the reason for the auto-fluorescence and its differences from tetracycline-fluorescence remain elusive. Most likely, a combination of various components is involved. One might argue that what was assumed to be the tetracycline-fluorescence over the last decade was indeed a mixture of both tetra- and auto-fluorescence of the bone.

Therefore, we have hypothesized that the loss in bone fluorescence is not absolutely correlated to alterations of the extracellular calcified osteoid matrix. Instead, we have postulated that the phenomenon of auto-fluorescence in healthy bone is primarily inherent to formations of bone collagen [13, 26]. We consider that one reason for fluorescence loss in necrotic bone is the successive destruction of collagen-forming structures much like in autofluorescence properties of soft tissues [27]. Furthermore, the various stages of collagen decline might be an explanation for the different fluorescence gradings in the observed auto-fluorescence.

In a case report with a histopathologic evaluation, Giovannacci et al. have noted our hypothesis and confirmed that the molecular source of the auto-fluorescence phenomena is linked to specific amino acids of the collagen molecules. They conclude that healthy bone strongly auto-fluoresces, whereas necrotic bone loses auto-fluorescence and appears much darker [28].

Indeed, an urgent need has existed for further basic research to investigate the fluorescence properties of bone and their differences under diverse conditions. This can only be performed in a preclinical model, since large bone blocks, as are necessary for such investigations, are rare. To overcome such difficulties, we have applied our established large animal model for osteonecrosis of the jaw [14–16] (i) to establish auto-fluorescence in healthy bone (no auto-fluorescence in necrotic bone), (ii) to investigate the histopathological setup and reason for auto-fluorescence, and (iii) to compare the macroscopic and histological differences between auto-fluorescence and tetracycline-fluorescence.

As expected the results of this preclinical trial demonstrate that, neither in vivo nor in vitro, could any macroscopic clinical differences in fluorescence be found between the auto-fluorescence and the tetracycline-fluorescence groups. Macroscopically, viable bone was marked by green fluorescence when visualized with a VELscope Vx® fluorescence lamp. In contrast, necrotic bone showed no or only pale fluorescence in both groups. To confirm the macroscopic results by histopathologic findings, we analyzed necrotic, non-necrotic, and control bone sections. Again, the fluorescence characteristics of the various bone sites were qualitatively evaluated and described for both study groups. The semi-quantitative and descriptive analyses of the necrotic and healthy signs correlated with the macroscopic clinical bone sections, and the macroscopic bone fluorescence confirmed the primary outcome of the study: necrosis occurred in the macroscopically non-fluorescent areas in the auto-fluorescence and tetracycline-fluorescence groups (no difference between the two groups). Moreover, healthy bone was present in the fluorescence areas in the auto-fluorescence and the tetracycline fluorescence groups (no difference between the two groups expected).

In the second part of the study, an evaluation was performed on both non-decalcified and decalcified histological preparations to determine whether the auto-fluorescence was mineral-dependent as assumed for tetracycline-fluorescence. As shown in the results of the semi-quantitative analysis, the decalcification of the sections decreased overall fluorescence. This is in line with the collagen-dependent fluorescence hypothesis. On decalcification, the stability and order of the collagen structure are destroyed, and therefore, the major fluorescence fades in paraffin sections. The remaining fluorescence is attributable to the bone cells (which contribute less brightness). In areas of necrosis with empty bone lacunae, no fluorescence can be detected at all.

**Fig. 6:**
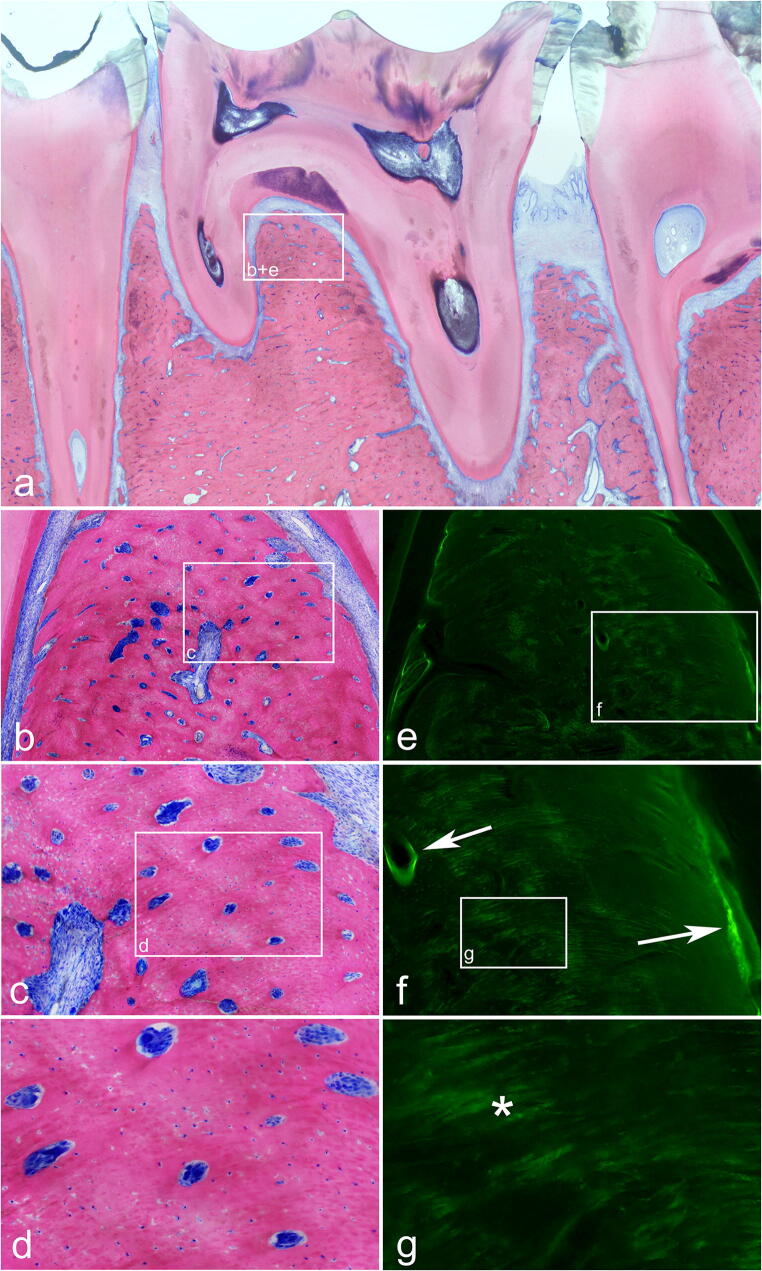
TF group, control side: **a** overview of the histopathologic preparation of healthy bone stock; Giemsa-Eosin-stained section. **b**–**f** Corresponding sections in detail; native and fluorescence. **e–g** Note that the viable bone is again marked by ordered collagen bands and bone cells (corresponding to the microscopic view of the auto-fluorescence group). Arrow: fluorescence is mainly caused by ordered collagen-bands. (*) Tetracycline fluorescence bands at the edge areas with new bone formations. Overall, no histologic difference can be seen between those two techniques

Interestingly, in a comparison between auto-fluorescence and tetracycline-fluorescence, no histologic difference was found between the two techniques. This agrees with the in vitro and the macroscopic in vivo results of this project. In some cases, stronger fluorescence bands appeared at the edge areas where new bone formations were being built in the tetracycline-fluorescence specimens (Fig. 6). However, this was not reproduceable throughout all sections (possibly because of the small number of animals). Overall, fluorescence in the tetracycline-fluorescence group was identically induced by the bone cells and by the ordered collagen structure. Therefore, a blinded and clear discrimination between the two techniques (auto- vs. tetracycline-fluorescence) was neither macroscopically nor microscopically possible. Indeed, what we initially thought to be tetracycline-fluorescence was instead a mixture of tetracycline (at the bone edges with increased bone formation) together with large components of auto-fluorescence. This agrees with the hypothesis suggested by the clinical studies that we have performed.

Spectral image acquisitions might be of interest in order to determine whether the qualitatively evaluated differences of auto- and tetracycline fluorescence are quantitatively divergent. This technique might help to quantify the characteristic fluorescence spectra and their different brightness. However, as discussed above, auto-fluorescence always severely impairs such investigations, and therefore, the authors believe that, even if the fluorescence color looked slightly different to the human eye, the results of spectral image acquisition have no further importance.

Interestingly, multiple necrotic lesions were found in the control areas of the contra-lateral upper and lower jaw in which no tooth extractions had been performed. Macroscopically and histologically, we found food entrapment that seemed to provoke local inflammation, leading to early necrotic bone changes in those areas. This is remarkable as inflammatory processes are known to play a vital role in the pathogenesis of MRONJ [29]. Poor oral hygiene and a relationship between oral surgical interventions with consecutive infectious processes have been mentioned as increasing the odds of MRONJ development. However, the evidence that gingivitis or periodontitis plays a role in the etiology of MRONJ is limited, although local infection and acidification are vital aspects of these pathologies [30, 31]. Despite the high frequency of periodontal disease, little data is currently available showing that periodontitis is an independent risk factor for MRONJ [32–34]. Scientific debate continues about the acidity in periodontal crevices, and both acidic and alkaline values have been reported [35]. Therefore, whether the inflammatory process induced by periodontitis is strong enough to cause MRONJ remains unknown. Further studies will be necessary investigate any associations between periodontal disease and the etiology of MRONJ with respect to the theory that infection and acidification are vital aspects in its pathogenesis.

The limitation of this present preclinical study is the small sample size evaluated. However, because of the preliminary proof-of-concept character, animal welfare and cost benefit relations, sample size was chosen for power estimations of larger trials in the future. Therefore, we tried our best to mainly create qualitative and semi-quantitative analysis, requiring fewer animals to give confidence that this method is reliably working. Indeed, conclusions drawn need to be carefully interpreted. A further possible drawback of the technique is the clinical application in the operating theater in a sterile setting. At present, simultaneous fluorescence visualization and bone ablation are not possible. The surgeon must change intermittently. In addition, bleeding in the operating area makes the fluorescence properties more difficult. Further developments to visualize the fluorescence freehand in order to osteotomized at the same time (e.g., with a fluorescence microscope) could improve the application in the future. Further studies will be necessary to investigate such improvements.

## Conclusion

Neither in vivo nor in vitro macroscopically differences are apparent between the auto-fluorescence and the tetracycline-fluorescence of bone. Non-fluorescent areas appear to be necrotic, whereas fluorescent areas are considered to represent viable bone (in the auto-fluorescence and tetracycline-fluorescence groups). No pure tetracycline-fluorescence areas occur. Indeed, the tetracycline-fluorescence is a mixture of tetracycline (at the bone edges with increased bone formation) and large components of auto-fluorescence. Causal for the auto-fluorescence are the arrangements and structure of collagen and the cell-filled bone lacunae.
